# Supplementary material for: Attitude toward vaccination against COVID-19 and acceptance of the national “QazVac” vaccine in the Aktobe city population, West Kazakhstan: A cross-sectional survey
Source: PLoS One. 2024 May 16;19(5):e0303854. doi: 10.1371/journal.pone.0303854 (PMC11098484; doi:10.1371/journal.pone.0303854)
Supplement: S1 Table — (DOCX) [file pone.0303854.s001.docx]

**Table S1**. **The English version of the Questionnaire to evaluate the attitude toward vaccination and acceptance of the “QazVac” vaccine against COVID-19 in the West Kazakhstan population.**

| Dear respondent!  This questionnaire was developed by the Department of Epidemiology of the West Kazakhstan Marat Ospanov Medical University and managed within the scientific project of republican importance, "The effect of vaccination on immunity in patients with the illness following COVID-19 among the population of Kazakhstan."  Your participation is voluntary, and your answers, whatever they are, do not imply restrictions on receiving clinical care determined by specialists.  We guarantee that all information you provide in the questionnaire is strictly confidential. Your data will be placed anonymously in the research database without mentioning personal details, i.e., using coded numbers. Your personal information will not be disclosed when publishing the results of this survey.  If you agree to participate as a subject of investigation, please check the consent box and fill out the informed consent form sent to your chat. Thus, you also consent to publishing the findings of this survey. You should return this form before or during the questionnaire filling process, which will take about 7-10 minutes.  Thank you in advance for participating in the survey! | | |
| --- | --- | --- |
| № | Question | Answer |
| 1. | Age | - 18-29 - 30-39 - 40-59 - 60-75 |
| 2. | Gender | - Male - Female |
| 3. | Level of education | - Incomplete secondary - Secondary education - Secondary special education - Higher education |
| 4. | Type of activity (occupation) | - Nonworking (unemployed, housewifes) - Student - Employed - Retired |
| 5. | How do you estimate your socioeconomic situation? (by monthly income per capita) | - High (500 thousand tenges and above) - Middle (from 100 to 500 thousand tenges) - Low (100 thousand tenges and below) |
| 6. | Do you have any chronic diseases | - Yes  - No |
| 6.1 | Arterial hypertension | - Yes - No |
| 6.2 | Coronary heart disease, angina pectoris | - Yes - No |
| 6.3. | Diabetes mellitus | - Yes - No |
| 6.4 | Others (inscribe) |  |
| 7. | What type of vaccine against COVID-19 did you receive last time? |  |
| 7.1 | - Sputnik V | - Yes - No |
| 7.2 | - QazVac | - Yes - No |
| 7.3 | - Hayat-Vax (Sinopharm) | - Yes - No |
| 7.4 | - CoronaVac (Sinovac) | - Yes - No |
| 7.5 | - BioNTech/Pfizer | - Yes - No |
| 7.6 | - I don’t remember the name of the vaccine | - Yes - No |
| 7.7 | - I didn’t take any | - Yes - No |
| 8. | The reason for getting vaccinated |  |
| 8.1 | - At my own will | - Yes - No |
| 8.2 | - At employer's request | - Yes - No |
| 8.3 | - Based on the recommendation of medical workers | - Yes - No |
| 8.4 | - Refused vaccination | - Yes - No |
| 9. | Why have you refused vaccination if yes? | - I'm afraid of complications after vaccination - I am against all vaccines - I do not believe in the effectiveness of the COVID-19 vaccine - I have medical contraindications - My religion does not allow it - I received the vaccine |
| 10. | Which sources of information do you trust? | - Official sources (media, medical professionals) - Unofficial sources (family members, colleagues) - Others (speeches of famous personalities, social networks – WhatsApp, YouTube) |
| 11 | In your opinion, has enough information about COVID-19 vaccination been provided in official sources? | - Few - Sufficient - Many - A vast amount of information |
| 12. | Which COVID-19 vaccine do you trust? |  |
| 12.1 | I do not trust all vaccines | - Yes - No |
| 12.2 | National vaccine (QazVac ) | - Yes - No |
| 12.3 | Neighboring countries (Sputnik V) | - Yes  - No |
| 12.4 | Far-abroad countries (Hayat-Vax, CoronaVac, BioNTech/Pfizer) | - Yes   - No |
| 12.5 | I trust all vaccines | - Yes   - No |
| 13. | Your attitude toward vaccination against COVID-19 | - Positive - Indifferent - Negative |
| 14. | What is your level of confidence in Kazakhstan's QazVac vaccine? | - I do not trust - I trust partially - I trust - I trust completely |
| 15. | Have you been ill with COVID-19? | - No - Not sure (I was more likely ill) - Yes |
| 16. | By what method of testing have you been diagnosed with COVID-19 disease? |  |
| 16.1 | PCR test | - Yes - No |
| 16.2 | ELISA for antibodies | - Yes - No |
| 16.3 | Chest X-ray / CT scan | - Yes - No |
| 16.4 | During the doctor's examination | - Yes - No |
| 17. | In what kind of condition did you suffer from the COVID-19 disease? | - Was not ill - Mild form - Moderate - Severe |
| 18. | Have you been ill with COVID-19 disease after vaccination? | - I have not received the vaccine - I was not ill - Not sure (more likely ill) - I was ill |
| 19 | How long have you been observed by a doctor after suffering COVID-19? | - Not observed by a doctor - 3 months - 6 months - 12 months (1 year) or more |
| 20. | Which of the following symptoms did you have after the disease? | - Yes - No |
| 20.1 | Fatigue | - Yes - No |
| 20.2 | Shortness of breath | - Yes - No |
| 20.3 | Joint pain | - Yes - No |
| 20.4 | Cough | - Yes - No |
| 20.5 | Violation of the sense of smell or taste | - Yes - No |
| 20.6 | Decreased memory, mental performance | - Yes - No |
| 20.7 | Depression | - Yes - No |
| 20.8 | Sleep disorder | - Yes - No |
| 20.9 | Rash | - Yes - No |
| 20.10 | Hair fall | - Yes - No |
| 20.11 | Allergy | - Yes - No |
| 20.12 | Others (inscribe) | - |
