# Supplementary material for: Attitude toward vaccination against COVID-19 and acceptance of the national “QazVac” vaccine in the Aktobe city population, West Kazakhstan: A cross-sectional survey
Source: PLoS One. 2024 May 16;19(5):e0303854. doi: 10.1371/journal.pone.0303854 (PMC11098484; doi:10.1371/journal.pone.0303854)
Supplement: S2 Table — (DOCX) [file pone.0303854.s002.docx]

**Table S2. Logistic regression analysis on relationships between the amount of information about COVID-19 vaccination in official sources and the vaccination status of respondents.**

| **Summary report on observations** | | | | | | | | | | | | | | | | | | | | | | | | | | | | | |  |  |  |  |
| --- | --- | --- | --- | --- | --- | --- | --- | --- | --- | --- | --- | --- | --- | --- | --- | --- | --- | --- | --- | --- | --- | --- | --- | --- | --- | --- | --- | --- | --- | --- | --- | --- | --- |
| Unweighed observations^a^ | | | | | | | | | | | | | | | | | | | N | | | | | % | | | | | |  |  |  |  |
| Selected observations | | | | | | | Included in analysis | | | | | | | | | | | | 2009 | | | | | 100,0 | | | | | |  |  |  |  |
|  |  |  |  |  |  |  | Missing observations | | | | | | | | | | | | 0 | | | | | ,0 | | | | | |  |  |  |  |
|  |  |  |  |  |  |  | Total | | | | | | | | | | | | 2009 | | | | | 100,0 | | | | | |  |  |  |  |
| Unselected observations | | | | | | | | | | | | | | | | | | | 0 | | | | | ,0 | | | | | |  |  |  |  |
| Total | | | | | | | | | | | | | | | | | | | 2009 | | | | | 100,0 | | | | | |  |  |  |  |
| a. If weighting is used, see the classification table for the total number of observations. | | | | | | | | | | | | | | | | | | | | | | | | | | | | | |  |  |  |  |
| **Coding of the dependent variable** | | | | | | | | | | | |  |  |  |  |  |  |  |  |  |  |  |  |  |  |  |  |  |  |  |  |  |  |
| Original value | | | | | Internal value | | | | | | |  |  |  |  |  |  |  |  |  |  |  |  |  |  |  |  |  |  |  |  |  |  |
| 0 | | | | | 0 | | | | | | |  |  |  |  |  |  |  |  |  |  |  |  |  |  |  |  |  |  |  |  |  |  |
| 1 | | | | | 1 | | | | | | |  |  |  |  |  |  |  |  |  |  |  |  |  |  |  |  |  |  |  |  |  |  |
| **Coding of categorical variables:** | | | | | | | | | | | | | | | | | | | | | | | | | | | | | | |  |  |  |
|  | | | | | | | | | | Frequency | | | | | Parameter encoding | | | | | | | | | | | | | | | |  |  |  |
|  |  |  |  |  |  |  |  |  |  |  |  |  |  |  | (1) | | | | | | (2) | | | | | (3) | | | | |  |  |  |
| 11.In your opinion, has enough information about COVID-19 vaccination been provided in official sources? | | | | | | | 1 | | | 408 | | | | | ,000 | | | | | | ,000 | | | | | ,000 | | | | |  |  |  |
|  |  |  |  |  |  |  | 2 | | | 1357 | | | | | 1,000 | | | | | | ,000 | | | | | ,000 | | | | |  |  |  |
|  |  |  |  |  |  |  | 3 | | | 125 | | | | | ,000 | | | | | | 1,000 | | | | | ,000 | | | | |  |  |  |
|  |  |  |  |  |  |  | 4 | | | 119 | | | | | ,000 | | | | | | ,000 | | | | | 1,000 | | | | |  |  |  |
| **Classification table^a,b^** | | | | | | | | | | | | | | | | | | | | | | | | | | | | | | |  |  |  |
|  | | Observed | | | | | | | | | Predicted | | | | | | | | | | | | | | | | | | | |  |  |  |
|  | |  |  |  |  |  |  |  |  |  | Vaccine+- | | | | | | | | | | | | | | Percent correct | | | | | |  |  |  |
|  | |  |  |  |  |  |  |  |  |  | 0 | | | | | | | 1 | | | | | | |  |  |  |  |  |  |  |  |  |
| Step 0 | | Vaccine+- | | | 0 | | | | | | 0 | | | | | | | 340 | | | | | | | ,0 | | | | | |  |  |  |
|  |  |  |  |  | 1 | | | | | | 0 | | | | | | | 1669 | | | | | | | 100,0 | | | | | |  |  |  |
|  |  | Total percentage | | | | | | | | |  | | | | | | |  | | | | | | | 83,1 | | | | | |  |  |  |
| a.Constant included in model. | | | | | | | | | | | | | | | | | | | | | | | | | | | | | | |  |  |  |
| b. Cutoff value - ,500 | | | | | | | | | | | | | | | | | | | | | | | | | | | | | | |  |  |  |
| **Variables in the equation** | | | | | | | | | | | | | | | | | | | | | | | | | | | | | | | | | |
|  | | | | B | | | | MSE | | | | | | | | | Wald | | | | | | DF | | | | sign. | | | | | Exp (B) | |
| Step 0 | | Constant | | 1,591 | | | | ,060 | | | | | | | | | 715,014 | | | | | | 1 | | | | ,000 | | | | | 4,909 | |
| **Variables missing from the equation** | | | | | | | | | | | | | | | | | | | | | | | | | | | | | | | | | |
|  | | | | | | | | | | | | | | | | | | | | | | | | Value | | | | | DF | | | | Sign. |
| Step 0 | | Variables | | 11. In your opinion, has enough information about COVID-19 vaccination been provided in official sources? | | | | | | | | | | | | | | | | | | | | 19,505 | | | | | 3 | | | | ,000 |
|  |  |  |  | 11. In your opinion, has enough information about COVID-19 vaccination been provided in official sources?(1) | | | | | | | | | | | | | | | | | | | | 17,223 | | | | | 1 | | | | ,000 |
|  |  |  |  | 11. In your opinion, has enough information about COVID-19 vaccination been provided in official sources?(2) | | | | | | | | | | | | | | | | | | | | ,043 | | | | | 1 | | | | ,835 |
|  |  |  |  | 11. In your opinion, has enough information about COVID-19 vaccination been provided in official sources? (3) | | | | | | | | | | | | | | | | | | | | 4,988 | | | | | 1 | | | | ,026 |
|  |  | Total Statistics | | | | | | | | | | | | | | | | | | | | | | 19,505 | | | | | 3 | | | | ,000 |
| **Universal criteria for model coefficients** | | | | | | | | | | | | | | | | | | | |  |  |  |  |  |  |  |  |  |  |  |  |  |  |
|  | | | Chi-Sqr. | | | | | | DF | | | | | sign. | | | | | |  |  |  |  |  |  |  |  |  |  |  |  |  |  |
| Step 1 | | Step | 18,595 | | | | | | 3 | | | | | ,000 | | | | | |  |  |  |  |  |  |  |  |  |  |  |  |  |  |
|  |  | Block | 18,595 | | | | | | 3 | | | | | ,000 | | | | | |  |  |  |  |  |  |  |  |  |  |  |  |  |  |
|  |  | Model | 18,595 | | | | | | 3 | | | | | ,000 | | | | | |  |  |  |  |  |  |  |  |  |  |  |  |  |  |
| **Summary for model** | | | | | | | | | | | | | | | | | | | |  |  |  |  |  |  |  |  |  |  |  |  |  |  |
| Step | -2 Log-plausibility | | | | | Cox and Snell R-square | | | | | | | Nagelkerke R-square | | | | | | |  |  |  |  |  |  |  |  |  |  |  |  |  |  |
| 1 | 1808,296^a^ | | | | | ,09 | | | | | | | ,15 | | | | | | |  |  |  |  |  |  |  |  |  |  |  |  |  |  |
| a. Evaluation stopped at iteration 4 as parameter estimates changed by less than .001. | | | | | | | | | | | | | | | | | | | |  |  |  |  |  |  |  |  |  |  |  |  |  |  |
| **Classification table^a^** | | | | | | | | | | | | | | | | | | | | | | | | | | | |  |  |  |  |  |  |
|  | | Observed | | | | | | | | | Predicted | | | | | | | | | | | | | | | | |  |  |  |  |  |  |
|  | |  |  |  |  |  |  |  |  |  | Vaccine+- | | | | | | | | | | | Percent correct | | | | | |  |  |  |  |  |  |
|  | |  |  |  |  |  |  |  |  |  | 0 | | | | | 1 | | | | | |  |  |  |  |  |  |  |  |  |  |  |  |
| Step 1 | | Vaccine+- | | | 0 | | | | | | 0 | | | | | 340 | | | | | | ,0 | | | | | |  |  |  |  |  |  |
|  |  |  |  |  | 1 | | | | | | 0 | | | | | 1669 | | | | | | 100,0 | | | | | |  |  |  |  |  |  |
|  |  | Total percentage | | | | | | | | |  | | | | |  | | | | | | 83,1 | | | | | |  |  |  |  |  |  |
| a. Cutoff value - ,500 | | | | | | | | | | | | | | | | | | | | | | | | | | | |  |  |  |  |  |  |

Reference value - Few.

| **Variables in the equation** | | | | | | | | | |
| --- | --- | --- | --- | --- | --- | --- | --- | --- | --- |
|  | | B | MSE | Wald | DF | sign. | Exp (B) | 95% Confidence interval for EXP(B) | |
|  |  |  |  |  |  |  |  | Lower | Upper |
| Step 1^a^ | 11. In your opinion, has enough information about COVID-19 vaccination been provided in official sources? |  |  | 19,195 | 3 | ,000 |  |  |  |
|  | 11. In your opinion, has enough information about COVID-19 vaccination been provided in official sources? (1) | ,539 | ,141 | 14,547 | 1 | ,000 | 1,714 | 1,300 | 2,261 |
|  | 11. In your opinion, has enough information about COVID-19 vaccination been provided in official sources? (2) | ,310 | ,263 | 1,386 | 1 | ,239 | 1,363 | ,814 | 2,283 |
|  | 11. In your opinion, has enough information about COVID-19 vaccination been provided in official sources? (3) | -,101 | ,244 | ,173 | 1 | ,678 | ,904 | ,560 | 1,458 |
|  | Константа | 1,234 | ,118 | 108,496 | 1 | ,000 | 3,435 |  |  |
| a. Variables entered in the step 1: 11. In your opinion, has enough information about COVID-19 vaccination been provided in official sources? | | | | | | | | | |
