# Supplementary material for: Attitude toward vaccination against COVID-19 and acceptance of the national “QazVac” vaccine in the Aktobe city population, West Kazakhstan: A cross-sectional survey
Source: PLoS One. 2024 May 16;19(5):e0303854. doi: 10.1371/journal.pone.0303854 (PMC11098484; doi:10.1371/journal.pone.0303854)
Supplement: S3 Table — (DOCX) [file pone.0303854.s003.docx]

**Table S3. Analysis of the relationship between attitudes toward vaccination and the other parameters, *N* 2,009.**

| **Parameters/**  **Items** | **Pearson’s**  **χ2** | ***N* 2,009** | **Treated positively (1,298)** | **Treated indifferently or negatively, *N* 711 (92+619)** | **P-value** |
| --- | --- | --- | --- | --- | --- |
| Gender | χ2 0.13 | Men:  643 (32%)  Women:  1,366 (68%) | 395 (30.4%)  903 (69.6%) | 248 (34.9%)  463 (65.1%) | 0.091 |
| Age | χ2 43.3 | 18-29:  972 (48.4%)  30-39:  428 (21.3%)  40-59:  403 (20.1%)  60-75:  206 (10.3%) | 795 (61.2%)  251 (19.3%)  174 (13.4%)  78 (6.1%) | 177 (24.9%)  177 (24.9%)  229 (32.2%)  128 (18%) | 0.001 |
| Education | χ2 22.6 | Incomplete secondary:  42 (2.1%)  Secondary (school):  335 (16.7%)  Secondary special:  361 (18%)  College:  1,271 (63.3%) | 21 (1.6%)  201 (15.5%)  235 (18.1%)  841 (64.8%) | 21 (2.9%)  134 (18.8%)  126 (17.8%)  430 (60.5%) | 0.001 |
| Income level | χ2 3.158 | High  299 (14.9%)  Middle  1,311 (65.3%)  Low  399 (19.9%) | 184 (14.2%)  865 (66.6%)  249 (19.2%) | 115 (16.2%)  446 (62.7%)  150 (21.1%) | 0.21 |
| Occupation | χ2 9.79 | Nonworking:  175 (8.7%)  Student:  936 (46.6%)  Employed:  825 (41.1%)  Retired:  73 (3.6%) | 99 (7.6%)  628 (48.4%)  530 (40.8%)  41 (3.2%) | 76 (10.7%)  308 (43.3%)  295 (41.5%)  32 (4.5%) | 0.02 |
| Presence of somatic chronic diseases | χ2 6.689 | Presence of chronic diseases  268 (13.3%)  Absence of chronic diseases  1,741 (86.7%) | 192 (14.8%)  1,106 (85.2%) | 76 (10.7%)  635 (89.3%) | 0.01 |
| Trust in different sources of information | χ2 14.92 | Official sources  1,125 (56%)  Unofficial sources  418 (20.8%)  Others  466 (23.2%) | 832 (66.4%)  256 (19.7%)  210 (13.9%) | 293 (45.3%)  162 (22.8%)  256 (31.9%) | 0.022 |
| Amount of information about vaccination in official sources | χ2 88.8 | Few  408 (20.3%)  Sufficient  1,357 (67.5%)  Many  125 (6.2%)  A vast amount of information  119 (5.9) | 191 (14.7%)  965 (74.3%)  79 (6.1%)  63 (4.9%) | 217 (30.5%)  392 (55.1%)  46 (6.5%)  56 (7.9%) | 0.001 |
| What is your level of confidence in Kazakhstan's QazVac vaccine? | χ2 449.5 | I do not trust  1,251 (62.3%)  I trust:  758 (37.7%) | 588 (45.3%)  710 (54.7%) | 663 (93.2%)  48 (6.8%) | 0.001 |
| Have you been ill with COVID-19? (History of COVID-19) | χ2 5.5 | No  1,233 (61.4%)  Not sure (was likely ill)  374 (18.6%)  Yes  402 (20.0%) | 795 (61.2%)  258 (19.9%)  245 (18.9%) | 438 (61.6%)  116 (16.3%)  157 (22.1%) | 0.064 |
