# Supplementary material for: Attitude toward vaccination against COVID-19 and acceptance of the national “QazVac” vaccine in the Aktobe city population, West Kazakhstan: A cross-sectional survey
Source: PLoS One. 2024 May 16;19(5):e0303854. doi: 10.1371/journal.pone.0303854 (PMC11098484; doi:10.1371/journal.pone.0303854)
Supplement: S4 Table — (DOCX) [file pone.0303854.s004.docx]

**Table S4. Analysis of the relationship between the history and severity of COVID-19 disease and trust in vaccines, *N* 2,009.**

| **Parameters/**  **Items** | ***N* 2,009** | **Those who trust all vaccines**  **(1,390)** | **Those who do not trust**  **(619)** | **Pearson’s**  **χ2** | **P-value** |
| --- | --- | --- | --- | --- | --- |
| The history and severity of COVID-19 | Was not ill  1,233 (61.3%)  Mild form  371 (18.5%)  Moderate form  341 (17%)  Severe form  64 (3.2%) | 854 (61.4%)  250 (18.0%)  238 (17.1%)  48 (3.5%) | 379 (61.2%)  121 (19.5%)  103 (16.6%)  16 (2.7%) | χ2 1.64 | 0.65 |
