# Supplementary material for: Attitude toward vaccination against COVID-19 and acceptance of the national “QazVac” vaccine in the Aktobe city population, West Kazakhstan: A cross-sectional survey
Source: PLoS One. 2024 May 16;19(5):e0303854. doi: 10.1371/journal.pone.0303854 (PMC11098484; doi:10.1371/journal.pone.0303854)
Supplement: S5 Table — (DOCX) [file pone.0303854.s005.docx]

**Table S5. Analysis of the relationship between trust in the “QazVac” vaccine and the other parameters, *N* 2,009.**

| **Parameters/**  **Items** | **Pearson’s χ2** | ***N* 2,009** | **Those who trust**  **(758)** | **Those who do not trust**  **(1,251)** | **P-value** |
| --- | --- | --- | --- | --- | --- |
| Gender | χ2 26.19 | Men:  643 (32%)  Women:  1,366 (68%) | 399 (52.7%)  359 (47.3%) | 244 (19.5%)  1,007 (80.5%) | 0.001 |
| Age | χ2 24.36 | 18-29:  972 (48.4%)  30-39:  428 (21.3%)  40-59:  403 (20.1%)  60-75:  206 (10.3%) | 266 (35.1%)  274 (36.1%)  125 (16.5%)  93 (12.3%) | 706 (56.4%)  129 (10.3%)  303 (24.3%)  113 (9.0%) | 0.004 |
| Education | χ2 4.35 | Incomplete secondary:  42 (2.1%)  Secondary (school):  335 (16.7%)  Secondary special:  361 (18%)  College:  1,271 (63.3%) | 20 (2.6%)  97 (12.8%)  110 (22.6%)  531 (62.1%) | 22 (2.8%)  238 (14.1%)  225 (23.6%)  740 (59.5%) | 0.23 |
| Income level | χ2 0.71 | High  299 (14.9%)  Middle  1,311 (65.3%)  Low  399 (19.9%) | 116 (15.3%)  486 (64.1%)  156 (20.6%) | 183 (14.6%)  825 (65.9%)  243 (19.4%) | 0.70 |
| Occupation | χ2 2.84 | Nonworking:  175 (8.7%)  Students:  936 (46.6%)  Employed:  825 (41.1%)  Retired:  73 (3.6%) | 57 (7.5%)  365 (48.2%)  307 (40.5%)  29 (3.8%) | 118 (9.4%)  571 (45.6%)  518 (41.4%)  44 (3.5%) | 0.42 |
| Presence of somatic chronic diseases | χ2 3.14 | Presence of chronic diseases  268 (13.3%)  Absence of chronic diseases  1,741 (86.7%) | 104 (13.7%)  654 (86.3%) | 164 (13.5%)  1,087 (86.5%) | 0.071 |
| Trust in different sources of information | χ2 11.44 | Official sources  1,125 (56%)  Unofficial sources  418 (20.8%)  Others  466 (23.2%) | 530 (69.9%)  135 (17.8%)  93 (12.2%) | 595 (47.6%)  283 (22.6%)  373 (21.8%) | 0.018 |
| Amount of information about vaccination in official sources | χ2 46.01 | Few  408 (20.3%)  Enough  1,357 (67.5%)  Many  125 (6.2%)  A vast amount of information  119 (5.9) | 96 (12.7%)  569 (75.1%)  51 (6.7%)  42 (5.5%) | 312 (24.9%)  788 (63.0%)  74 (5.9%)  77 (6.2%) | 0.001 |
| Have you been ill with COVID-19? (History of COVID-19) | χ2 0.365 | No  1,233 (61.4%)  Not sure (was likely ill)  374 (18.6%)  Yes  402 (20.0%) | 463 (61.1%)  146 (19.3%)  149 (19.7%) | 770 (61.6%)  228 (18.2%)  253 (20.2%) | 0.83 |
