# Supplementary material for: Attitude toward vaccination against COVID-19 and acceptance of the national “QazVac” vaccine in the Aktobe city population, West Kazakhstan: A cross-sectional survey
Source: PLoS One. 2024 May 16;19(5):e0303854. doi: 10.1371/journal.pone.0303854 (PMC11098484; doi:10.1371/journal.pone.0303854)
Supplement: S6 Table — (DOCX) [file pone.0303854.s006.docx]

**Table S6.** **Logistic regression analysis on relationship between the amount of information about COVID-19 vaccination in official sources and the trust in the domestic “QazVac” vaccine.**

| **Summary report on observations** | | | | | | | | | | | | | | | | | | | | | | | | | | | | | | |  |  |  |
| --- | --- | --- | --- | --- | --- | --- | --- | --- | --- | --- | --- | --- | --- | --- | --- | --- | --- | --- | --- | --- | --- | --- | --- | --- | --- | --- | --- | --- | --- | --- | --- | --- | --- |
| Unweighed observations^a^ | | | | | | | | | | | | | | | | | | | | N | | | | | % | | | | | |  |  |  |
| Selected observations | | | | | | | | | Included in analysis | | | | | | | | | | | 2009 | | | | | 100,0 | | | | | |  |  |  |
|  |  |  |  |  |  |  |  |  | Missing observations | | | | | | | | | | | 0 | | | | | ,0 | | | | | |  |  |  |
|  |  |  |  |  |  |  |  |  | Total | | | | | | | | | | | 2009 | | | | | 100,0 | | | | | |  |  |  |
| Unselected observations | | | | | | | | | | | | | | | | | | | | 0 | | | | | ,0 | | | | | |  |  |  |
| Total | | | | | | | | | | | | | | | | | | | | 2009 | | | | | 100,0 | | | | | |  |  |  |
| a. If weighting is used, see the classification table for the total number of observations. | | | | | | | | | | | | | | | | | | | | | | | | | | | | | | |  |  |  |
| **Coding of the dependent variable** | | | | | | | | | | | | | |  |  |  |  |  |  |  |  |  |  |  |  |  |  |  |  |  |  |  |  |
| Original value | | | | | | Internal value | | | | | | | |  |  |  |  |  |  |  |  |  |  |  |  |  |  |  |  |  |  |  |  |
| 0 | | | | | | 0 | | | | | | | |  |  |  |  |  |  |  |  |  |  |  |  |  |  |  |  |  |  |  |  |
| 1 | | | | | | 1 | | | | | | | |  |  |  |  |  |  |  |  |  |  |  |  |  |  |  |  |  |  |  |  |
| **Coding of categorical variables:** | | | | | | | | | | | | | | | | | | | | | | | | | | | | | | | |  |  |
|  | | | | | | | | | | | | Frequency | | | | | Parameter encoding | | | | | | | | | | | | | | |  |  |
|  |  |  |  |  |  |  |  |  |  |  |  |  |  |  |  |  | (1) | | | | | | (2) | | | | | (3) | | | |  |  |
| 11.In your opinion, has enough information about COVID-19 vaccination been provided in official sources? | | | | | | | | | 1 | | | 408 | | | | | ,000 | | | | | | ,000 | | | | | ,000 | | | |  |  |
|  |  |  |  |  |  |  |  |  | 2 | | | 1357 | | | | | 1,000 | | | | | | ,000 | | | | | ,000 | | | |  |  |
|  |  |  |  |  |  |  |  |  | 3 | | | 125 | | | | | ,000 | | | | | | 1,000 | | | | | ,000 | | | |  |  |
|  |  |  |  |  |  |  |  |  | 4 | | | 119 | | | | | ,000 | | | | | | ,000 | | | | | 1,000 | | | |  |  |
| **Classification table^a,b^** | | | | | | | | | | | | | | | | | | | | | | | | | | | | | | | | | |
|  | | Observed | | | | | | | | | | | | | | | | | Predicted | | | | | | | | | | | | | | |
|  | |  |  |  |  |  |  |  |  |  |  |  |  |  |  |  |  |  | 12.2 National ( QazVac ) | | | | | | | | | | | | | Percent correct | |
|  | |  |  |  |  |  |  |  |  |  |  |  |  |  |  |  |  |  | 0 | | | | | | | 1 | | | | | |  |  |
| Step 0 | | 12.2 National vaccine ( QazVac ) | | | | | | | | | | | 0 | | | | | | 1251 | | | | | | | 0 | | | | | | 100,0 | |
|  |  |  |  |  |  |  |  |  |  |  |  |  | 1 | | | | | | 758 | | | | | | | 0 | | | | | | ,0 | |
|  |  | Total percentage | | | | | | | | | | | | | | | | |  | | | | | | |  | | | | | | 62,3 | |
| a.Constant included in model. | | | | | | | | | | | | | | | | | | | | | | | | | | | | | | | | | |
| b. Cutoff value - ,500 | | | | | | | | | | | | | | | | | | | | | | | | | | | | | | | | | |
| **Variables in the equation** | | | | | | | | | | | | | | | | | | | | | | | | | | | | | | | | | |
|  | | | | | B | | | | | MSE | | | | | | | | | Wald | | | | | DF | | | | | sign. | | | | Exp (B) |
| Step 0 | | Constant | | | -,501 | | | | | ,046 | | | | | | | | | 118,481 | | | | | 1 | | | | | ,000 | | | | ,606 |
| **Variables missing from the equation** | | | | | | | | | | | | | | | | | | | | | | | | | | | | | | | | | |
|  | | | | | | | | | | | | | | | | | | | | | Value | | | | | | DF | | | | | sign. | |
| Step 0 | | Variables | | | | | | 11. In your opinion, has enough information about COVID-19 vaccination been provided in official sources? | | | | | | | | | | | | | 46,013 | | | | | | 3 | | | | | ,000 | |
|  |  |  |  |  |  |  |  | 11. In your opinion, has enough information about COVID-19 vaccination been provided in official sources? (1) | | | | | | | | | | | | | 31,402 | | | | | | 1 | | | | | ,000 | |
|  |  |  |  |  |  |  |  | 11. In your opinion, has enough information about COVID-19 vaccination been provided in official sources? (2) | | | | | | | | | | | | | ,535 | | | | | | 1 | | | | | ,465 | |
|  |  |  |  |  |  |  |  | 11. In your opinion, has enough information about COVID-19 vaccination been provided in official sources? (3) | | | | | | | | | | | | | ,320 | | | | | | 1 | | | | | ,572 | |
|  |  | Total Statistics | | | | | | | | | | | | | | | | | | | 46,013 | | | | | | 3 | | | | | ,000 | |
| **Universal criteria for model coefficients** | | | | | | | | | | | | | | | | | | | | | |  |  |  |  |  |  |  |  |  |  |  |  |
|  | | | | Chi-Sqr. | | | | | | | DF | | | | | sign. | | | | | |  |  |  |  |  |  |  |  |  |  |  |  |
| Step 1 | | Step | | 48,381 | | | | | | | 3 | | | | | ,000 | | | | | |  |  |  |  |  |  |  |  |  |  |  |  |
|  |  | Block | | 48,381 | | | | | | | 3 | | | | | ,000 | | | | | |  |  |  |  |  |  |  |  |  |  |  |  |
|  |  | Model | | 48,381 | | | | | | | 3 | | | | | ,000 | | | | | |  |  |  |  |  |  |  |  |  |  |  |  |
| **Summary for model** | | | | | | | | | | | | | | | | | | | | | |  |  |  |  |  |  |  |  |  |  |  |  |
| Step | -2 Log-plausibility | | | | | | Cox and Snell R-square | | | | | | | | Nagelkerke R-square | | | | | | |  |  |  |  |  |  |  |  |  |  |  |  |
| 1 | 2614,460^a^ | | | | | | ,24 | | | | | | | | ,32 | | | | | | |  |  |  |  |  |  |  |  |  |  |  |  |
| a. Evaluation stopped at iteration 4 as parameter estimates changed by less than .001. | | | | | | | | | | | | | | | | | | | | | |  |  |  |  |  |  |  |  |  |  |  |  |
| **Classification table^a^** | | | | | | | | | | | | | | | | | | | | | | | | | | | | | | | | | |
|  | | | Observed | | | | | | | | | | | | | | | Predicted | | | | | | | | | | | | | | | |
|  | | |  |  |  |  |  |  |  |  |  |  |  |  |  |  |  | Homeland QazVac+- | | | | | | | | | | | | Percent correct | | | |
| **Classification table^a^** | | | | | | | | | | | | | | | | | | | | | | | | | | | | | | | | | |
| Step 1 | | 12.2 National ( QazVac ) | | | | | | | | | | | 0 | | | | | | 1251 | | | | | | | 0 | | | | | | 100,0 | |
|  |  |  |  |  |  |  |  |  |  |  |  |  | 1 | | | | | | 758 | | | | | | | 0 | | | | | | ,0 | |
|  |  | Total percentage | | | | | | | | | | | | | | | | |  | | | | | | |  | | | | | | 62,3 | |
| a. Cutoff value - ,500 | | | | | | | | | | | | | | | | | | | | | | | | | | | | | | | | | |

Reference value - Few:

| **Variables in the equation** | | | | | | | | | |
| --- | --- | --- | --- | --- | --- | --- | --- | --- | --- |
|  | | B | MSE | Wald | DF | sign. | Exp (B) | 95% Confidence interval for EXP(B) | |
|  |  |  |  |  |  |  |  | Lower | Upper |
| Step 1^a^ | 11. In your opinion, has enough information about COVID-19 vaccination been provided in official sources? |  |  | 44,491 | 3 | ,000 |  |  |  |
|  | 11. In your opinion, has enough information about COVID-19 vaccination been provided in official sources? (1) | ,853 | ,129 | 43,709 | 1 | ,000 | 2,347 | 1,822 | 3,022 |
|  | 11. In your opinion, has enough information about COVID-19 vaccination been provided in official sources? (2) | ,806 | ,216 | 13,912 | 1 | ,000 | 2,240 | 1,466 | 3,422 |
|  | 11. In your opinion, has enough information about COVID-19 vaccination been provided in official sources? (3) | ,573 | ,225 | 6,501 | 1 | ,011 | 1,773 | 1,142 | 2,753 |
|  | Constant | -1,179 | ,117 | 101,986 | 1 | ,000 | ,308 |  |  |
| a. Variables entered in the step 1: 11. In your opinion, has enough information about COVID-19 vaccination been provided in official sources?. | | | | | | | | | |
