# Supplementary material for: Attitude toward vaccination against COVID-19 and acceptance of the national “QazVac” vaccine in the Aktobe city population, West Kazakhstan: A cross-sectional survey
Source: PLoS One. 2024 May 16;19(5):e0303854. doi: 10.1371/journal.pone.0303854 (PMC11098484; doi:10.1371/journal.pone.0303854)
Supplement: S7 Table — (DOCX) [file pone.0303854.s007.docx]

**Table S7. Analysis of the relationship between the disease after vaccination and attitudes toward vaccination, *N* 1,669.**

| **Parameters/**  **Items** | **Pearson’s**  **χ2** | ***N* 1,669** | **Treated**  **vaccination positively**  **(1,188)** | **Treated indifferently or negatively**  **(481)** | **P-value** |
| --- | --- | --- | --- | --- | --- |
| Have you been ill with COVID-19 disease after vaccination? | χ2 7,73 | I was not ill  1,210 (72.5%)  Not sure  287 (17.2%)  I was ill  172 (10.3%) | 884 (74.4%)  192 (16.2%)  112 (9.4%) | 326 (67.8%)  95 (19.8%)  60 (12.5%) | 0.021 |
