# Supplementary material for: Attitude toward vaccination against COVID-19 and acceptance of the national “QazVac” vaccine in the Aktobe city population, West Kazakhstan: A cross-sectional survey
Source: PLoS One. 2024 May 16;19(5):e0303854. doi: 10.1371/journal.pone.0303854 (PMC11098484; doi:10.1371/journal.pone.0303854)
Supplement: Attachment — Submitted filename: Comments to Reviewer 2.docx [file pone.0303854.s008.docx]

**Comments to Reviewer 2:**

Dear Reviewer! We highly appreciate the time and effort you spent analyzing our article. We are very touched by the warmth of your comments regarding our work. We have made the necessary changes as you recommended. Revision notes include point-by-point responses to your concerns and comments (all inserts are colored in red.)

**Comments from Reviewer 1 and our responses:**

The paper investigates the attitudes towards vaccination against COVID-19 and the level of confidence in the domestic QazVac vaccine in Aktobe city, West Kazakhstan, by means of a cross-sectional study. The results highlight some individual characteristics more correlated with the choice to get vaccinated. This is an important and emerging topic and it is focused in a area less covered by scholars. In my opinion these are good points to consider the paper to be published. Nevertheless, the manuscript needs some revisions in order to be suitable for POEN journal. In the following lines I will express my main concerns on the paper. I hope that my comments can be helpful for the author(s).

Recommended suggestions

*1. In the abstracts (in the method subsection) I would suggest to add more information on the quantitative methodology used in the paper*

- Dear Reviewer! Regrettably, we failed to insert our methods and statistical tests due to word restrictions. Our abstract took almost 300 words according to the journal requirements but had undergone substantial changes:

The scale of emergency caused by COVID-19, the ease of survey, and the crowdsourcing deployment guaranteed by the latest technology have allowed unprecedented access to data describing behavioral changes induced by the pandemic. The study aimed to present the survey results to identify attitudes toward vaccination against COVID-19 among the population of West Kazakhstan, the level of confidence in the national QazVac vaccine, and the role of different sources of information on COVID-19 in decision-making concerning vaccination. A computer-assisted survey was conducted using WhatsApp messenger. Overall, 2,009 participants responded, with a response rate of 92%. Most (83.1%) were immunized against COVID-19; among them, 20.1% obeyed the request of their employers that had been practiced within non-pharmaceutical interventions to contain the disease. The youngest respondents, individuals with a college education, students, and employed people, as well as those with chronic diseases, showed positive attitudes toward vaccination (all p˂0.05). About two-thirds of respondents (69.2%) expressed trust in all types of vaccines against COVID-19. Of those who refused vaccination (16.9%), about one-third feared vaccination consequences, and more than a third (38.2%) reported anti-vaccine sentiments. The decisive factors in accepting vaccination were trust in official sources of information (reports of medical experts, etc.) and, mainly, subjectively interpreted sufficiency of information about the disease, which had increased the odds of being vaccinated by 63.9% (OR 1.71, 95% CI [1.3;2.26], p<0.05). Confidence in the domestic QazVac vaccine was expressed by 37.7% of respondents. History and severity of COVID-19 disease did not play a role in positive perceptions of vaccination, while illness after vaccination substantially affected vaccination approval (p 0.021). No significant differences have been observed regarding the overall performance across five vaccines (QazVac, Sputnik V, CoronaVac, Hayat-Vax, and BioNTech/Pfizer) available for Kazakhstan's population (p 0.27).

We may have devoted a disproportionate amount of space to the findings.

*2. The organization of the paper (in terms of sections sequence) is a little bit strange and unusual for me. The authors highlight the main findings of previous studies in section 4. I think that a background section after the brief introduction is needed (before data, methodology and results). In the results section the authors can make comments on the consistence between their results and previous results.*

- We completely agree with you, dear Reviewer! The previous organization of the article's idea was extremely awkward. The introduction was concise and did not match the article's content to a definite extent. We have substantially worked out all the article's sections to bring them into logical correspondence, and we placed the main comments in the Discussion section. We did not design a Background section as there were no previous results. The present article is the first within the ongoing project on vaccination issues in Kazakhstan.

*3. In the introduction section the authors should also briefly highlight that vaccination is one of the main policies aimed at reduce the risk of contagions. In fact, this is a pharmaceutical measure that has been supported also by non-pharmaceutical interventions (NPI). Please note that there are also some studies specifically focused on central Asia. I suggest some paper that could be probably useful in order to better frame this policy intervention among the public health policies chosen by national governments.
• Alfano, V. (2022). COVID-19 in Central Asia: exploring the relationship between governance and non-pharmaceutical intervention. Health Policy and Planning, 37(8), 952-962.
• Alfano, V., Ercolano, S., & Pinto, M. (2023). Modeling Central Asia’s Choices in Containing COVID-19: A Multivariate Study. Administration & Society, 55(9), 1819-1836.
• Perra, N. (2021). Non-pharmaceutical interventions during the COVID-19 pandemic: A review. Physics Reports, 913, 1-52.
• Mendez-Brito, A., El Bcheraoui, C., & Pozo-Martin, F. (2021). Systematic review of empirical studies comparing the effectiveness of non-pharmaceutical interventions against COVID-19. Journal of Infection, 83(3), 281-293.*

- Dear Reviewer! We are very grateful for your invaluable assistance in improving the presentation of our data. We used these papers as references in the Introduction section, making the section more comprehensive and reflecting the modern concept of the pandemic and its control.

*4. The factors able to influence the choice to get vaccinated need to be better discussed. There are several papers with good literature review. I suggest the following papers just as starting point to be discussed in the background section
• Alfano, V., & Ercolano, S. (2022). Your vaccine attitude determines your altitude. What are the determinants of attitudes toward vaccination?. Vaccine, 40(48), 6987-6997.
• Baumgaertner B, Carlisle JE, Justwan F, Rabinowitz M. The influence of political ideology and trust on willingness to vaccinate. PLoS ONE 2018;13(1): e0191728.
• Czarnek G, Kossowska M, Szwed P. Political ideology and attitudes toward vaccination: A study report. Working paper; 2020
• Fridman A, Gershon R, Gneezy A, Capraro V. COVID-19 and vaccine hesitancy: A longitudinal study. PLoS ONE 2021;16(4):e0250123.
• Gessner BD, Kaslow D, Louis J, Neuzil K, O’Brien KL, Picot V, et al. Estimating the full public health value of vaccination. Vaccine 2017;35(46):6255–63.
• Hak E, Schönbeck Y, De Melker H, Van Essen GA, Sanders EA. Negative attitude of highly educated parents and health care workers towards future vaccinations in the Dutch childhood vaccination program. Vaccine 2005;23 (24):3103–7.
• Lazarus JV, Wyka K, Rauh L, Rabin K, Ratzan S, Gostin LO, et al. Hesitant or not? The association of age, gender, and education with potential acceptance of a COVID-19 vaccine: a country-level analysis. J Health Commun 2020;25 (10):799–807.
• Lewandowsky S, Gignac GE, Oberauer K, Denson T. The role of conspiracist ideation and worldviews in predicting rejection of science. PLoS ONE 2013;8 (10):e75637.
• Maurer J, Uscher-Pines L, Harris KM. Perceived seriousness of seasonal and A (H1N1) influenzas, attitudes toward vaccination, and vaccine uptake among US adults: does the source of information matter? Prev Med 2010;51 (2):185–7.*

- Dear Reviewer! All these papers were thoroughly read and utilized both in the Introduction and the Discussion sections, enriching the content of our very simple article designed exclusively to present the results. Owing to your contribution, the article has been upgraded impressively. The philosophic content of these remarkable papers you kindly shared with us extends beyond the issues of pandemic control. I am personally thankful to you for such a present.

*5. On the basis of the suggested literature, I think that the authors can improve the discussion and the interpretation of their results.*

- Undoubtedly! I think, dear Reviewer, you can see for yourself by reviewing the present version of the manuscript.

*6. The paper is focused on the vaccine hesitancy that represent an important issue correlated with the aim of the manuscript. Nevertheless, in my opinion the authors should also consider other approach more based on the attitudes toward vaccination (that is probably more focused on their data). In fact, as pointed out by Alfano and Ercolano, the attitudes toward vaccination could represent a more complex and complete rationale rather than the simple hesitancy. In fact, if we measure individual attitude we can range from hesitancy to positive attitude toward vaccination.*

- Dear Reviewer! In the revised article's version, more attention is paid to attitudes towards vaccination, and we examined this attitude in two planes based on the same material. We analyzed our sample from the perspective of vaccination status and then without it, i.e., from a person's free will. There were differences, although they appeared to be small. It means that a person can get vaccinated by obeying the employer's request (in our article, there are 20.1% of such individuals), but his (she's) way of thinking may vary significantly, up to anti-vaccine sentiments. Unfortunately, the format of the article and questionnaire (20 questions in total, 6 of them - demographics, and 5 - experience of the disease) did not allow us to go beyond the immediate needs. It would be very interesting to analyze more deeply those who refused vaccination. Our main conclusion evidences the dominating role of information sources in the decision-making regarding vaccination (in a broad sense, not only against Covid-19). These findings are in line with the provisions of the Fridman's article.

*7. The sample used in the paper is a convenience sample. I think that the authors should discuss this point in the paper. Moreover it could be interesting to have some statistics in order to compare some caracteristics of the sample population with the whole population of Aktobe (age, gender, education).*

- Dear Reviewer! Regrettably, we failed to comply with your recommendation to compare our sample with the general sample of Aktobe. The only statistics we cited in the article are the number of Aktobe residents (549,381 as of 2022).

*8. Honestly, I am not fully convinced of the quantitative methodology used by authors. In my opinion, in order to have a good ceteris paribus correlations the authors should use a regression model as the related literature. This kind of methodology could help the authors to go ahead the definition of the social profile of the residents of Aktobe who has been vaccinated. This is a very important point in order to improve the robustness of the paper. In the current version we know just some bivariate correlations but if I understood the methodology, the ceteris paribus condition is not respected. The authors should try to address this concern.*

- We absolutely agree with you, dear Reviewer! We thoroughly remade the article's content, and deleted this irrelevant social profile. We used logistic regression and brought our findings in the Results section:

We utilized a simple binary logistic regression model to determine the predictors of favorable vaccination decisions. A total of 8 variables with p<0.1 were present in the univariate analysis (Table 1). The multivariate LRA of these variables showed that, after adjusting, only one variable (the amount of relevant information) contributed significantly to the participants’ willingness to be vaccinated (p-log<0.05). Our analysis presented a chi-square statistic (χ²(1) = 18.6, p<0.001), refuting the null hypothesis and signifying that the “sufficient” amount of available information predicts the decision to get a vaccine (Table S2). The model's R2 value, standing at 0.15, suggests that variations in the amount of information account for approximately 15% of the variance in the final decision, highlighting the influence of “sufficient” available information about the disease in official sources on the eventual decision of getting a vaccine in the respondents. The odds ratio (OR) calculated at 1.71, with a 95% CI of [1.3;2.26], p < 0.05, elucidates that a “sufficient” amount of relevant information increases the odds of being vaccinated by 63.9%. When the respondents assess the amount of information as “many” or “too many (excessive),” the odds of deciding on vaccination turn insignificant: OR 1.36, 95% CI of [0.81;2.28], p 0.24, and OR 0.9, 95% CI of [0.56;1.45], p 0.68, respectively. (lines 328-343)

And:

We designed a binary logistic regression model to reveal the possible predictors of trust in the national vaccine. A total of 5 variables with p<0.1 were present in the univariate analysis (Table S5). After adjusting for the other variables, the multivariate analyses resulted in the presence of one variable (the amount of relevant information) that contributed significantly to the participants’ trust in the national vaccine (p-log<0.05). A chi-square statistic χ²(1) of 48.4 p<0.001 signifies that “sufficient” information in official sources predicts trust in the domestic vaccine. The model’s R2 value of 0.32 suggests that variations in the amount of information account for approximately 32% of the variance in the final result, highlighting the influence of available information about the disease and a vaccine of interest in official sources on the judgment about the vaccine in the respondents. The OR 2.35, with a 95% CI of [1.82;3.02], p < 0.05, evidence that a “sufficient” amount of relevant information increases the odds of trust in the QazVac vaccine by 70.1%. Yet, the likelihood of confidence in the vaccine decreases with further increments up to the level of “many information” (OR 2.24, 95% CI [1.47;3.42], p 0.001) and turns insignificant at the level of “superfluous information” (OR 1.77, 95% CI [1.14;2.75], p 0.11). (lines 445-460)

9. I think that the authors should try to deepener the policy implication of their study.

- Dear Reviewer! We did our best owing to your references. Regrettably, it's a limit. As mentioned above, the discussion of issues of vaccination refusal or negative attitude is worth of the separate article. We will try to deepen this burning topic.

*Other minor suggestions (not compulsory)
10. I appreciated some technical tables in the supplemental material (like the questionaiires) but I think that the tables with the main results should be in the main text.*

- Dear Reviewer! We almost radically changed the article's content, replacing appropriate tables.

Thank you again for bringing all inconsistencies to our attention and for your constructive comments on assisting us in finding the revision points. We hope, after revisions, you find our manuscript more suitable for publication.
